# Resin acids as inducible chemical defences of pine seedlings against chewing insects

**DOI:** 10.1371/journal.pone.0232692

**Published:** 2020-05-01

**Authors:** Xosé López-Goldar, Lina Lundborg, Anna Karin Borg-Karlson, Rafael Zas, Luis Sampedro

**Affiliations:** 1 Department of Ecology and Evolutionary Biology, Cornell University, Ithaca, New York, United States of America; 2 Misión Biológica de Galicia (MBG-CSIC), Pontevedra, Galicia, Spain; 3 Department of Chemistry, School of Engineering Sciences in Chemistry, Biotechnology and Health, Royal Institute of Technology (KTH), Stockholm, Sweden; University of Richmond, UNITED STATES

## Abstract

Inducibility of defences in response to biotic stimuli is considered an important trait in plant resistance. In conifers, previous research has mostly focused on the inducibility of the volatile fraction of the oleoresin (mono- and sesquiterpenes), leaving the inducibility of the non-volatile resin acids largely unexplored, particularly in response to real herbivory. Here we investigated the differences in the inducibility of resin acids in two pine species, one native from Europe (*Pinus pinaster* Ait.) and another from North America (*Pinus radiata* D. Don), in response to wounding by two European insects: a bark chewer, the pine weevil (*Hylobius abietis* L.), and a defoliator, the pine processionary caterpillar (*Thaumetopoea pityocampa* Schiff.). We quantified the constitutive (control) and induced concentrations of resin acids in the stem and needles of both pine species by gas chromatography techniques. Both pine species strongly increased the concentration of resin acids in the stem after pine weevil feeding, although the response was greater in *P*. *pinaster* than in *P*. *radiata*. However, systemic defensive responses in the needles were negligible in both pine species after pine weevil feeding in the stem. On the other hand, *P*. *radiata* locally reduced the resin acid concentration in the needles after pine caterpillar feeding, whereas in *P*. *pinaster* resin acid concentration was apparently unaffected. Nevertheless, systemic induction of resin acids was only observed in the stem of *P*. *pinaster* in response to pine caterpillar feeding. In summary, pine induced responses were found highly compartmentalized, and specific to herbivore identity. Particularly, plant defence suppression mechanisms by the pine caterpillar, and ontogenetic factors might be potentially affecting the induced response of resin acids in both pine species.

## Introduction

Conifer terpenoids have myriad implications in plant defence [see reviews of 1, and 2] and ecological roles in plant-organism interactions [3, 4]. Terpenoids are the main components of the oleoresin, comprising a volatile fraction of mono- (C_10_) and sesquiterpenes (C_15_), and a non-volatile fraction of diterpenes (C_20_) [5]. Mono- and diterpenes are synthesized via the methylerythritol pathway in the plastid, whereas sesquiterpenes are synthesized via the mevalonate pathway in the cytosol [3, 6]. Mono- and sesquiterpenes have been widely studied and, due to their volatile nature, have relevant ecological roles in plant interactions with conspecifics and other organisms [2, 3]. Several studies reported a high specificity and direct dose-dependent toxic effects on herbivore and pathogens [7–10]. Diterpenes are also considered major chemical defences in conifers, particularly after subsequent hydroxylation and oxidation of neutral diterpenes by cytochrome P450 monooxygenases [11, 12], originating diterpene resin acids (hereafter ‘resin acids’). Previous works have tested the activity of resin acids against particular enemies by using known concentrations of individual purified compounds in the lab [13–17]. However, research on the defensive function of this chemical fraction of the oleoresin has comparatively received much less attention [see for example 18, 19–21] than that on the volatile fraction (mono- and sesquiterpenes).

Inducibility of defences in response to biotic stimuli is considered an important trait in plant resistance [21, 22]. Inducible responses may entail specific changes in the chemical profile and concentration of key secondary metabolites that may be contingent on the attacker identity [9, 23, 24]. Differences in the inducibility of secondary metabolites among genotypes, populations or species may explain differences in resistance against enemies [21, 22, 25]. Despite the continuous effort in understanding the variability of induced responses of secondary metabolites and its implication in biotic resistance in conifers [see for example 26, 27–29],little is still known about how inducibility of resin acids can vary depending on the herbivore attacker identity, and how these responses differ among pine species.

In spite of the existing body of knowledge about induced defences in pine trees, systemic induced responses in distal parts of the plant from the site of damage have been often overlooked [30, 31]. Biotic challenge can effectively induce local defensive responses–and hamper enemy entrance in the wounded zone–but might also impede further aggressions in distal parts of the plant through systemic induced resistance [32], or facilitated via associated systemic susceptibility [30, 33]. Systemic induced responses of conifer resin acids have been studied after fungal challenge [22], mechanical wounding [34] or chemical elicitation [35], but systemic responses to insect herbivory remain largely unexplored.

The objective of this study is to investigate the differences in the inducibility of the resin acid fraction in two pine species commonly used in forestry in Mediterranean areas, one native from Europe (*Pinus pinaster* Ait.) and another from North America (*Pinus radiata* D. Don), in response to two of the most harmful pest insect herbivores of conifers in Europe differing in their target tissue: a bark chewer, the pine weevil (*Hylobius abietis* L.), and a defoliator, the pine processionary caterpillar (*Thaumetopoea pityocampa* Schiff.). The pine weevil is widely distributed across Europe and Asia [36], causes severe damage on young conifer seedlings, and have a strong impact on plant fitness and early survival [37, 38]. The success in the regeneration of conifer forests by planting is highly contingent on this insect [37, 39], which causes important economic losses in large part of Europe [36]. The pine caterpillar is distributed across the Mediterranean region of southern Europe and North Africa and it is responsible for severe defoliation of young and adult trees of several pine species, with consequences of major loss in tree growth, and subsequent tree death in extreme infestations [40, 41]. Both insect herbivores showed sensitivity to conifer semiochemicals, with volatile terpenes mediating host selection for oviposition [42, 43] and insect orientation and preference [see for instance, 7], and providing antifeedant activity [see for example, 8, 21, 44]. In addition, specific induction of volatile terpenes was observed in response to damage inflicted by both insects, and those induced responses varied depending on the analysed plant tissue and the conifer host species [9, 45]. However, knowledge on the inducibility of resin acids in the interaction between pines and these herbivores remains unexplored. To this end, we performed a factorial experiment with herbivory (control, pine weevil and processionary caterpillar) and pine species as the main factors, and we studied the changes in the concentration and profile of resin acids in the needles and stem tissues. Results regarding mono- and sesquiterpenes and total phenolics have been reported elsewhere [9]. Here, we asked whether the inducibility of resin acids is i) different between pine species; ii) specific to the herbivore attacker, and iii) restricted to the target tissue where the insect feeds.

## Experimental

### Natural history

We focused on two pine species currently coexisting in Southern Europe. Maritime pine (*Pinus pinaster* Ait.) is a native species of the Iberian Peninsula, whose distribution ranges from South France and Southeast Europe, to North Africa. Monterey pine (*P*. *radiata* D. Don) has its origin in California and was introduced in Spain around 1840. Nowadays both species overlap in the Atlantic region of the Iberian Peninsula in a common altitude range from 0 to 800 m. a. s. l.

### Experimental design

We conducted a factorial greenhouse experiment with two main factors: pine species (*P*. *pinaster* and *P*. *radiata*) and real herbivory treatment for induction of plant defences (control, pine weevil feeding and pine caterpillar feeding). The experiment followed a randomized split-plot design replicated in 10 blocks, with herbivory treatment as the whole factor and pine species as the split factor. Sample size was 60 pine plants in total (3 induction treatments × 2 species × 10 blocks).

### Plant material

Pine seeds were treated with a fungicide (Fernide®, Syngenta Agro, Spain) to avoid interferences from pernicious pathogens and individually sown in 2 L pots using a mixture of perlite and peat as substrate (1:1, v:v) with 12 g of a slow-release fertilizer (Multicote®). Seedlings were grown in a glass greenhouse with controlled light conditions (minimum 12 h per day), and temperature (25°C day, 10°C night) and watered daily as described in Moreira et al. [9] until one year old (average pine height was 41.2 ± 2.4 cm for *P*. *pinaster* and 62.7 ± 4.0 cm for *P*. *radiata*). Then, all plants were carefully covered with a nylon mesh to avoid insect to escape and randomly allotted to the herbivory treatments (control, pine weevil and pine caterpillar).

### Herbivory treatments

Adult pine weevils were captured in the field during the summer of 2009 using Nordlander traps [46], kept in culture chambers at 15°C and fed with fresh pine twigs for two weeks. Before the weevil-induction treatment, weevils were starved for 48 h in Petri dishes with a moistened filter paper (15°C and dark) and then weighed. One specimen was placed on each young pine, allowed to feed for 5 days and then removed and weighed again.

Nests with pine caterpillars were collected in the field from infested trees during the summer of 2009, transported to the lab in ice-coolers, immediately opened and second instar larvae were gently separated. Groups of 10 caterpillars were starved as above for 12 h and weighed. Two groups of 10 caterpillars were placed with each plant, one on needles at the upper part and the other at the bottom part. Caterpillars were allowed to feed for 6 days and then removed, counted and weighed.

No insect died during the feeding period and all plants were damaged. Damage caused either by the pine weevils or caterpillars did not significantly differ between pine species [47].

### Sampling and chemical analysis

After herbivory-induction treatments, each plant was harvested by cutting the stem aboveground. A fresh 1.5 cm-long stem segment from the middle part and a sample of needles randomly chosen (approximately 0.2 g) were collected from each plant, weighed, flash frozen and preserved at -80°C in cryogenic vials for terpenoid analyses.

Extraction of terpenoids in the phloem and needles were performed following the procedure described in Sampedro et al. [48] with modifications. Samples were finely ground in Teflon tubes with liquid nitrogen and terpenes were extracted by using ultrapure n-hexane in an ultrasonic bath at 25°C. Aliquots of those extracts (500 μl) were dried under constant N_2_ flow, allowing mono- and sesquiterpenes to volatilize, and the remaining diterpenes and resin acids were resuspended in 750 μL of methanol using 0.1 mg·mL^-1^ heptadecanoic acid (#H3500, Sigma-Aldrich) as internal standard. Then, diterpene resin acids were derivatized to their methyl esters with 75 μL of tetramethylammonium hydroxide (#334901, Sigma-Aldrich).

Resin acids in the extracts were identified at KTH (Stockholm, Sweden) by gas chromatography-mass spectrometry (GC-MS) in total ion current (TIC) mode (scan range 40–400 m/z). The GC-MS system consisted of an Agilent HP6890 GC coupled with a 5793 MS. The separation was performed on a DB-5 capillary column (30 m, i. d. 0.25 mm, film thickness 0.25 μm, Agilent Technologies, CA, USA). A volume of 1 μL of each sample was injected in splitless mode, using Helium as carrier gas with a flow rate of 1 mL·min^-1^. The oven program was set at 152°C for 2 minutes, followed by a temperature ramp of 3°C·min^-1^ up to 260 and maintained at this temperature for 5 min. Injector temperature was set at 250°C isothermal. The peaks present in each TIC chromatogram (see S1 Fig) were identified by comparing their spectra to those in the NIST and Wiley Mass Spectra Libraries included in the software MSD Enhanced Chemstation software (version E.02.01.1177, Agilent Technologies, CA, USA) and in the literature [49], and comparing their calculated retention indices to those found in the literature [50].

Resin acids were quantified at MBG (Pontevedra, Spain) with a GC-Flame Ionization Detector (FID) Perkin Elmer Clarus 500 equipped with an Elite-5 capillary column (30 m, ID 0.25 mm, film thickness 0.25 μm, Perkin Elmer, MA, USA) equivalent to the previous DB-5. All GC separation parameters were configured identically to the previous analysis at KTH. Hydrogen was used as carrier gas. FID temperature was set at 300°C. Quantification of resin acids was performed by using a calibration curve of commercial standard of abietic acid (#00010, Fluka). Individual resin acid concentration was expressed as mg·g^-1^ dry weight (d. w.). Proportion of extracted resin acids was finally compared with that of previous research studies in the same pine species (only data for *P*. *pinaster* was available to date) that used similar or different solvents as ours, finding minor differences [21, 51–54]. This supports the reliability and reproducibility of our extraction procedure of resin acids.

Presence of univocally identified resin acids in at least three samples of the induction treatment (undamaged controls, pine weevil or pine caterpillar) was used as selection criteria before analysis. Nine resin acids were selected (S2 Fig). In the analytical separations, two main resin acids coeluted and were treated as one compound (levopimaric + palustric acids) in the statistical analyses.

### Statistical analysis

For each insect herbivore, effects of herbivory treatment on the concentration of total and individual resin acids in the stem and the needles of each pine species were analysed with a mixed model by restricted maximum likelihood (PROC MIXED procedure in SAS 9.4, Cary, NC). Induction treatment (T), pine species (SP) and their interaction (T × SP) were considered fixed factors. Block (B) and the interaction (B × T) were treated as random factors in order to test the main effect T with the appropriate error term [55]. Independent analyses were carried out for each insect herbivore and each tissue. Specific contrast tests were also performed for each pine species to study the effects of each insect herbivore in the concentration of total and individual resin acids for each pine tissue regarding control plants. Residual normality was achieved by log-transforming the raw data when necessary, and heterogeneous variance models for factor T were used when significantly improved model fit. Two cases were removed due to sampling mistakes. Results are shown as least square mean ± standard error (s. e.).

The multivariate changes in the profile and concentration of resin acids between species, herbivory treatments, and plant tissues were analysed by a Principal Component Analysis using PROC PRINCOMP in SAS 9.4. The main resin acids were summarized in the first two components, which were analysed to test the effects of T, SP and the T × SP interaction on the multivariate profile of resin acids by using the mixed model from above.

## Results

### Concentration of total resin acids in response to herbivory

**Fig 1:**
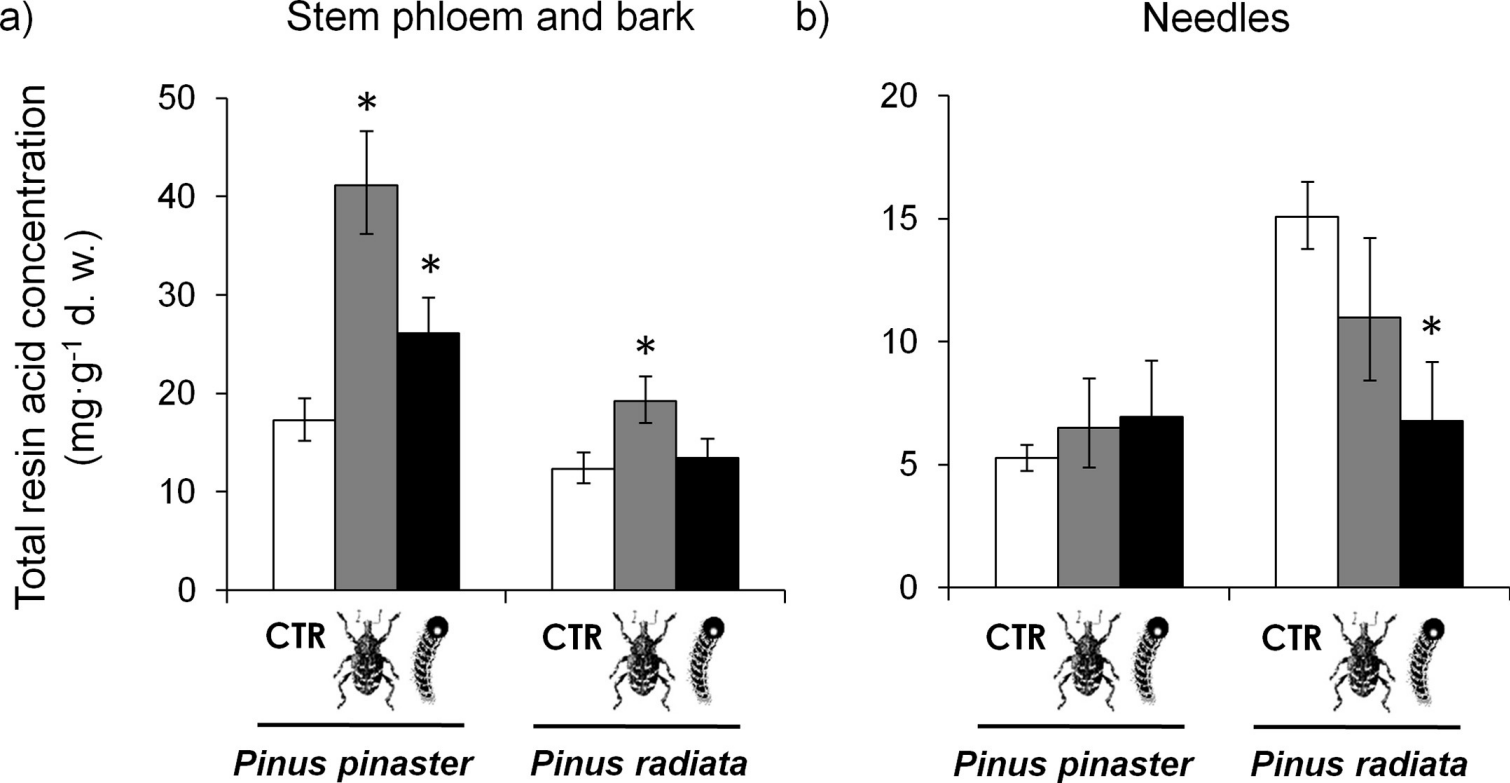
Local and systemic effects of damage by two chewing insect herbivores, the pine weevil (grey bars, a bark chewer) and the pine caterpillar (black bars, a defoliator) on the total concentration of resin acids in the stem phloem and bark (a) and in the needles (b) of pine seedlings of two species. LS means ± s.e. (N = 9–10). The asterisk above bars indicates significant differences with control plants within each species (*P* < 0.05).

The total concentration of resin acids significantly differed between pine species in both plant tissues (Table 1). Total concentration of resin acids in the stem was higher in *P*. *pinaster* than in *P*. *radiata*, whereas *P*. *radiata* showed higher concentration of resin acids in the needles than *P*. *pinaster* (Fig 1). Pine weevil feeding significantly increased the concentration of total resin acids in the stem of both pine species (Table 1, Fig 1A) but not in the needles (Table 1, Fig 1B). Local induction of total resin acids in the stem after weevil feeding was larger in *P*. *pinaster* than in *P*. *radiata* (Fig 1A). These differences in the induced response resulted in a marginally significant species × weevil interaction for the concentration of total resin acids in the stem (Table 1).

**Table 1:** Effects of the herbivore-induction treatments on the concentration of total resin acids in the stem (a) and in the needles (b) tissues of two pine species.

	Pine weevil effect			Pine caterpillar effect		
	*Df*	*F*	*P*	*Df*	*F*	*P*
*a) Stem*						
Species (SP)	1, 17	24.64	**<0.001**	1, 18	21.26	**<0.001**
Induction Treatment (T)	1, 9	27.11	**<0.001**	1, 9	2.69	0.135
SP × T	1, 17	4.00	0.062	1, 18	2.4	0.139
*b) Needles*						
Species (SP)	1, 18	17.73	**<0.001**	1, 17	6.31	**0.022**
Induction Treatment (T)	1, 9	0.09	0.770	1, 9	1.39	0.268
SP × T	1, 18	1.99	0.175	1, 17	6.9	**0.018**

Significant p-values (*P* < 0.05) are typed in bold.

Pine caterpillar defoliation did not affect the overall concentration of total resin acids neither in the stem nor in the needles across species (Table 1). However, an interesting and significant species × caterpillar interaction was found for total resin acids in the needles (Table 1). A 55% reduction of the concentration of resin acids after caterpillar feeding was found in *P*. *radiata* needles, but no clear response was detected in *P*. *pinaster* (Fig 1B). Specific contrasts within each species also indicated a significant systemic increase of total resin acids in the stems of *P*. *pinaster* after caterpillar feeding, but no response in *P*. *radiata* (Fig 1A).

### Concentration of individual resin acids in response to herbivory

**Fig 2:**
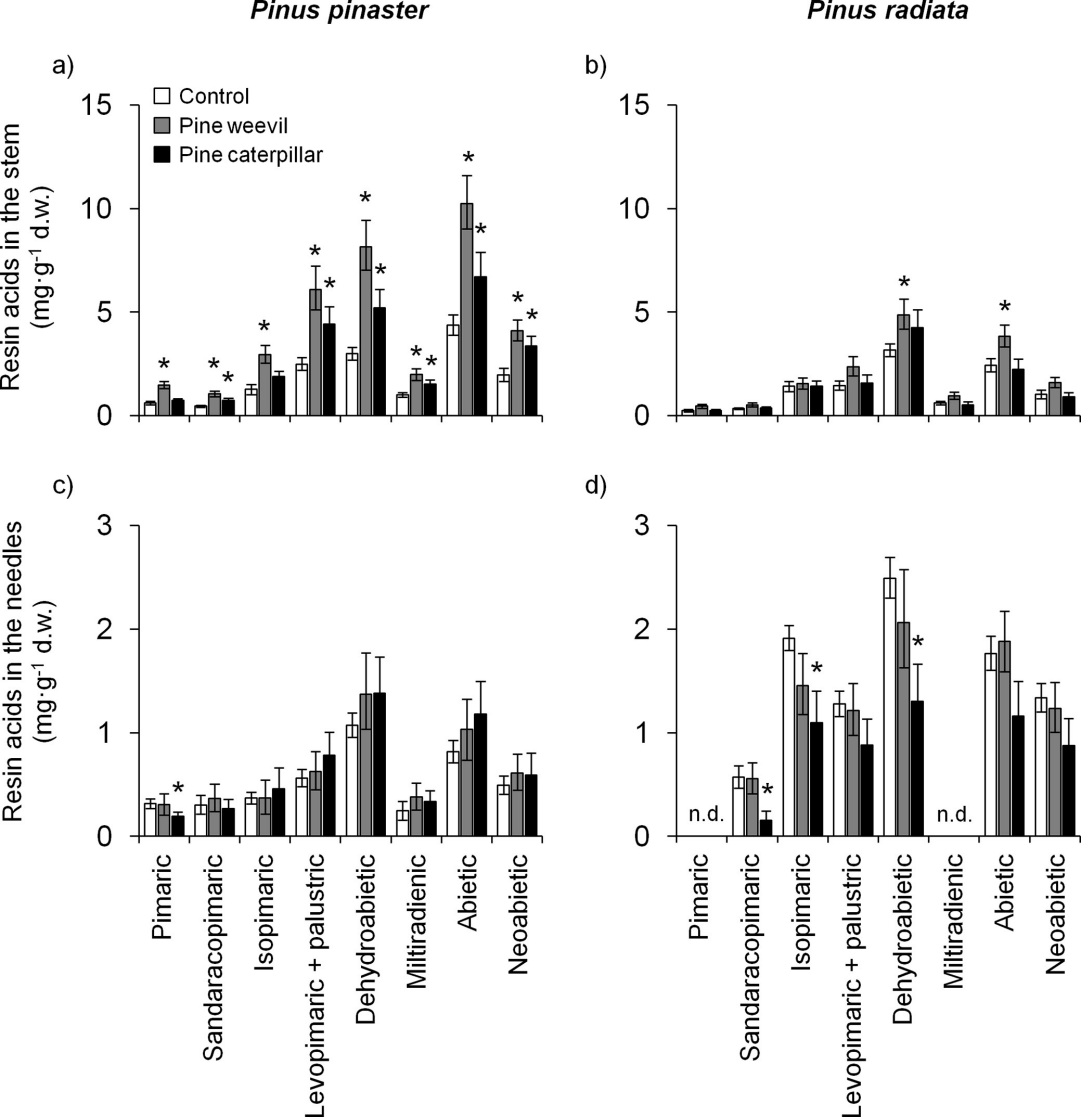
**Effects of herbivory-induction by pine weevil (grey bars) and pine caterpillar (black bars) on the concentration of individual resin acids in the stem phloem and in the needles of two pine species.** LS means ± s.e. (N = 9–10). The asterisk above bars indicates significant differences between herbivory treatment in regard to control plants for each compound within each species (*P* < 0.05). n.d. = not detected.

More than 95% of the total amount of diterpenes in the stem and needles extracts of the two pine species consisted of the resin acids presented in Fig 2. Pimaric acid was not detected in the needles of *P*. *radiata*, whereas only traces of miltiradienic acid were detected in *P*. *radiata* needles (Table 2). Statistical analyses of these two compounds in the needles were restricted to *P*. *pinaster*.

**Table 2:** Summary of the eight resin acids identified and quantified by GC-MS and GC-FID analyses, respectively, in the stem phloem and needles of 1-year-old seedlings of *Pinus pinaster* and *P*. *radiata*.

				*P*. *pinaster*		*P*. *radiata*	
Resin acid	RT *(min)*	KI_C_	KI_L_	Stem *Mean ± s*.*e (n)*	Needles *Mean ± s*.*e (n)*	Stem *Mean ± s*.*e (n)*	Needles *Mean ± s*.*e (n)*
Pimaric	23.619	2247	2237	0.60±0.11 (10)	0.32±0.10 (9)	0.25±0.09 (10)	– (0)
Sandaracopimaric	24.083	2264	2256	0.46±0.04 (10)	0.30±0.13 (9)	0.34±0.04 (10)	0.57±0.15 (7)
Isopimaric	25.205	2307	2297	1.19±0.23 (10)	0.37±0.06 (10)	1.35±0.25 (10)	1.91±0.12 (10)
Levopimaric + palustric	25.485	2315	2306	2.49±0.31 (10)	0.56±0.08 (10)	1.46±0.22 (10)	1.28±0.11 (10)
Dehydroabietic	26.367	2348	2341	2.89±0.29 (10)	1.07±0.11 (10)	3.09±0.31 (10)	2.49±0.19 (10)
Miltiradienic	26.838	2362	–	1.01±0.11 (10)	0.25±0.12 (7)	0.61±0.09 (10)	– (0)
Abietic	27.525	2390	2385	4.36±0.58 (10)	0.84±0.14 (10)	2.43±0.37 (10)	1.81±0.14 (10)
Neoabietic	28.959	2442	2443	1.96±0.28 (10)	0.49±0.08 (9)	1.03±0.19 (10)	1.33±0.13 (10)

Compounds were identified (as their methyl esters) using the NIST and Wiley Mass Spectral Libraries (%), and from the correspondence between the calculated Kovat’s Index (KI_C_) with those published in the literature when available [49, 50, KI_L_]. Compounds were quantified using abietic acid (as its methyl ester) as standard (Sigma-Aldrich). All resin acids accounted for > 95% of total amount of diterpenes. Shown are the constitutive concentrations (mg·g^-1^ dry weight, mean ± s.e.) and sample size (n) of each resin acid from the stem and needles of control plants for each of the pine species.

RT: retention time.

Concentrations of all resin acids in the stems, except isopimaric and dehydroabietic acids, were higher in *P*. *pinaster* than in *P*. *radiata* (Tables 3A and 4A, Fig 2A and 2B). However, *P*. *radiata* needles showed higher concentrations of all resin acids than *P*. *pinaster* needles, except for pimaric, sandaracopimaric, and miltiradienic acids (Tables 3B and 4B, Fig 2C and 2D).

**Table 3:** Effects of the pine weevil feeding on the concentration of the main resin acids in the stem (a) and in the needles (b) tissues of two pine species.

	Pine species			Pine weevil			Species × weevil		
	*Df*	*F*	*P*	*Df*	*F*	*P*	*Df*	*F*	*P*
*a) Stem*									
Pimaric	1,17	46.28	**<0.001**	1,9	23.85	**<0.001**	1,17	6.13	**0.024**
Sandaracopimaric	1,17	16.08	**<0.001**	1,9	24.27	**<0.001**	1,17	5.17	**0.036**
Isopimaric	1,17	3.37	0.084	1,9	9.23	**0.014**	1,17	6.3	**0.023**
Levopimaric+Palustric	1,17	23.42	**<0.001**	1,9	20.34	**0.002**	1,17	3.06	0.098
Dehydroabietic	1,17	4.29	0.054	1,9	31.79	**<0.001**	1,17	6.73	**0.019**
Miltiradienic	1,17	22.14	**<0.001**	1,9	18.81	**0.002**	1,17	2.08	0.167
Abietic	1,17	48.68	**<0.001**	1,9	23.0	**0.001**	1,17	4.62	**0.046**
Neoabietic	1,17	36.89	**<0.001**	1,9	20.95	**0.001**	1,17	2.85	0.109
b) *Needles*									
Pimaric	–	–	–	1,9	0.67	0.433	–	–	–
Sandaracopimaric	1,18	2.73	0.116	1,9	0.03	0.859	1,18	0.09	0.767
Isopimaric	1,18	57.41	**<0.001**	1,9	0.88	0.373	1,18	0.97	0.339
Levopimaric+Palustric	1,18	18.2	**<0.001**	1,9	0.0	0.951	1,18	0.18	0.674
Dehydroabietic	1,18	12.44	**0.002**	1,9	0.0	0.981	1,18	1.46	0.243
Miltiradienic	–	–	–	1,9	0.55	0.467	–	–	–
Abietic	1,18	19.01	**<0.001**	1,9	0.28	0.607	1,18	0.08	0.786
Neoabietic	1,18	24.46	**<0.001**	1,9	0.03	0.868	1,18	0.6	0.450

Significant p-values (*P* < 0.05) are typed in bold.

–: ‘Pine species’ and ‘Species × weevil’ effects were not computed due to low sample size/no detection of the resin acid in *Pinus radiata*. Thus, ‘Pine weevil’ effect was tested only for *P*. *pinaster* in those cases.

**Table 4:** Effects of the pine caterpillar feeding on the concentration of the main resin acids in the stem (a) and in the needles (b) tissues of two pine species.

	Pine species			Pine caterpillar			Species × caterpillar		
	*Df*	*F*	*P*	*Df*	*F*	*P*	*Df*	*F*	*P*
*a) Stem*									
Pimaric	1,18	36.63	**<0.001**	1,9	0.44	0.523	1,18	0.87	0.364
Sandaracopimaric	1,18	15.76	**<0.001**	1,9	3.65	0.088	1,18	3.87	0.065
Isopimaric	1,18	0.7	0.413	1,9	1.19	0.304	1,18	2.56	0.127
Levopimaric+Palustric	1,18	22.23	**<0.001**	1,9	3.99	0.077	1,18	2.90	0.106
Dehydroabietic	1,18	0.41	0.530	1,9	5.88	**0.038**	1,18	0.81	0.381
Miltiradienic	1,18	31.55	**<0.001**	1,9	1.07	0.327	1,18	4.69	**0.044**
Abietic	1,18	37.62	**<0.001**	1,9	1.34	0.277	1,18	3.88	0.065
Neoabietic	1,18	38.09	**<0.001**	1,9	2.22	0.170	1,18	5.14	**0.036**
b) *Needles*									
Pimaric	–	–	–	1,9	2.3	0.163	–	–	–
Sandaracopimaric	1,17	0.47	0.500	1,9	5.76	**0.040**	1,17	4.05	0.060
Isopimaric	1,17	32.75	**<0.001**	1,9	1.71	0.223	1,17	4.05	0.060
Levopimaric+Palustric	1,17	6.54	**0.020**	1,9	0.08	0.784	1,17	3.62	0.074
Dehydroabietic	1,17	5.87	**0.027**	1,9	1.47	0.256	1,17	7.53	**0.014**
Miltiradienic	–	–	–	1,9	0.08	0.789	–	–	–
Abietic	1,17	4.53	**0.048**	1,9	0.07	0.793	1,17	4.85	**0.042**
Neoabietic	1,14	11.11	**0.004**	1,9	0.53	0.487	1,17	2.4	0.140

Significant p-values (*P* < 0.05) are typed in bold.

–: ‘Pine species’ and ‘Species × caterpillar’ effects were not computed due to low sample size/no detection of the resin acid in *Pinus radiata*. Thus, ‘Pine caterpillar’ effect was tested only for *P*. *pinaster* in those cases.

Concentrations of all the individual resin acids in the stems were higher after pine weevil damage, but no significant changes were observed in the needles (Table 3). Induced resin acids in the stem were, in average, 1.4-fold and 0.6-fold higher than in the corresponding control plants of *P*. *pinaster* and *P*. *radiata*, respectively (Fig 2A and 2B). These quantitative differences in the induced responses between species were reflected in significant species × weevil interactions for most resin acids: pimaric, sandaracopimaric, isopimaric, dehydroabietic and abietic acids (Table 3A). Specific contrasts showed that all compounds were induced in the stems of *P*. *pinaster* (Fig 2A), and only dehydroabietic and abietic acids were induced in the stems of *P*. *radiata* after weevil feeding (Fig 2B). No significant species × weevil interactions were detected for the concentration of resin acids in the needles (Table 3B). All specific contrasts between control and weevil-induced plants indicated no significant systemic effect of weevil feeding on the concentration of individual resin acids in the needles of the two pine species (Fig 2C and 2D).

Specific contrasts showed that, after caterpillar feeding, most resin acids significantly increased their concentration in the stem of *P*. *pinaster* but not of *P*. *radiata* (Fig 2A and 2B). However, significant species × caterpillar interactions were only found for miltiradienic and neoabietic acids in the stem (Table 4A). Moreover, a general trend of reduction of resin acids in the needles in response to caterpillar feeding was found in *P*. *radiata* but not in *P*. *pinaster* (Fig 2C and 2D), despite the species × caterpillar interactions were only significant for dehydroabietic and abietic acids (Table 4B). Specific contrasts showed significant reductions in the concentration of sandaracopimaric (-72%), isopimaric (-43%) and dehydroabietic acids (-48%) after pine caterpillar feeding in the needles of *P*. *radiata* compared to control plants (Fig 2D). Weak responses were detected in *P*. *pinaster* needles. Pimaric acid was the only that showed a significant reduction (-39%) for this species after pine caterpillar herbivory (Fig 2C).

### Multivariate changes in the profile and concentration of resin acids in response to herbivory

**Fig 3:**
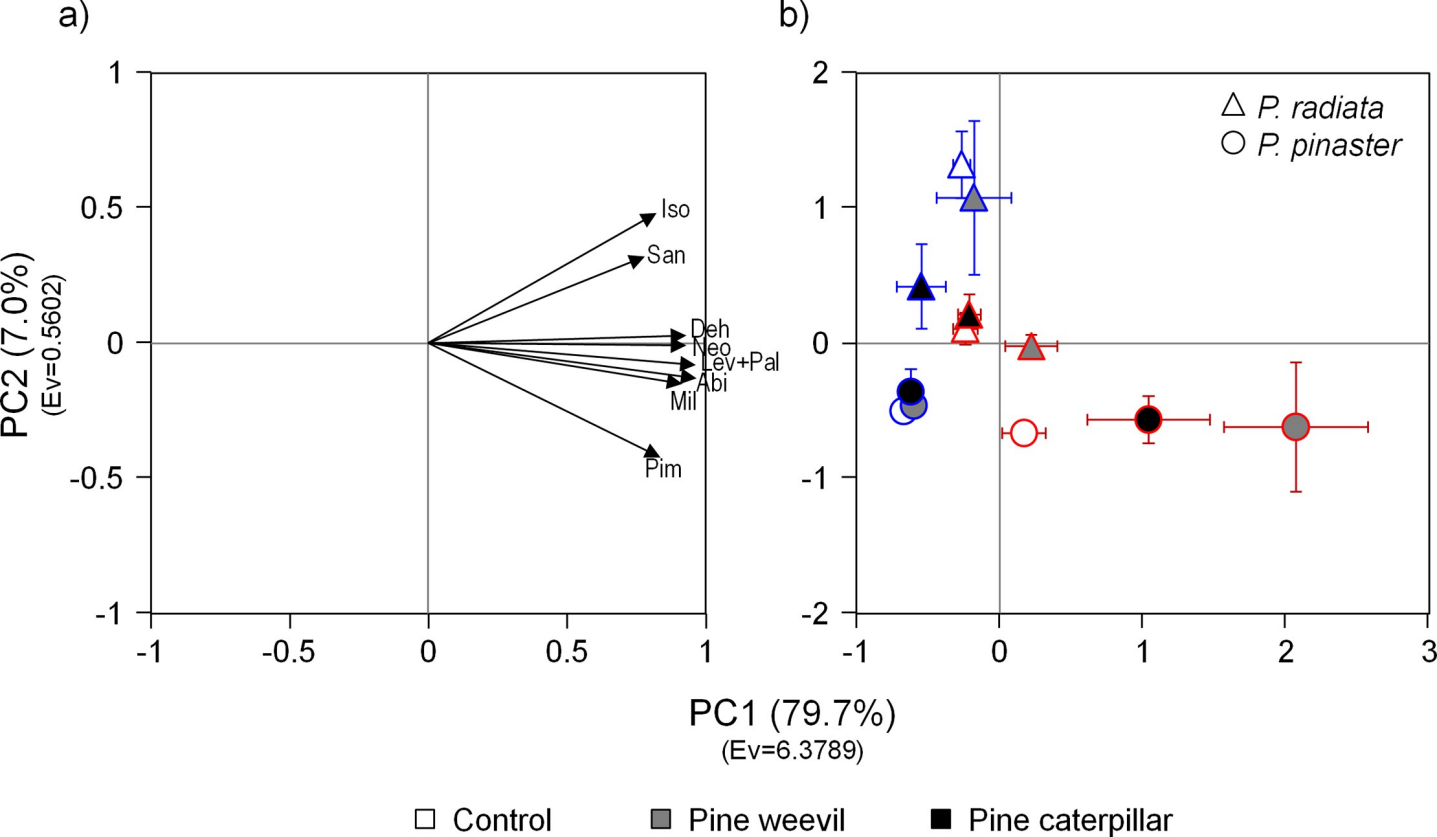
Principal component analysis of the constitutive concentration of the main resin acids and their induced responses in the stem phloem and needles after herbivory by two insect chewers. a) Graphical representation of the variable loadings for each component; b) chemical profile and concentration of resin acids in the multivariate space depicted by pine species, herbivory treatments and plant tissues (coloured symbol borders and error bars: red = stem phloem, blue = needles). Each symbol and error bars represent the mean of PC1 and PC2 and their standard errors, respectively, for each combination of pine species, herbivory treatments, and plant tissues (N = 9–10 plants). Variance explained (in %) and eigenvalue (Ev) of each component are indicated in brackets. Abi = abietic, Deh = dehydroabietic, Iso = isopimaric, Lev+Pal = levopimaric + palustric, Neo = neoabietic, Pim = pimaric, San = sandaracopimaric, Mil = miltiradienic.

PCA revealed contrasted patterns in the chemical profiles and concentrations of constitutive and induced resin acids (Fig 3). The first component (PC1) absorbed 79.7% of the total variance, and was strongly and positively related with the concentration of all resin acids (Fig 3A). The second component (PC2) explained 7.0% of the total variance, and was related to changes in the chemical profile, being positively related, in particular, with the concentrations of isopimaric and sandaracopimaric acids, and negatively related with the concentration of pimaric acid (Fig 3A). In the stems, *P*. *pinaster* showed greater constitutive values of PC1 and lower of PC2 than *P*. *radiata*, indicating high concentrations of resin acids with a pimaric-enriched profile (Fig 3B). In the needles, *P*. *radiata* showed greater constitutive values of PC1 and PC2 than *P*. *pinaster*, indicating high concentrations of resin acids, particularly enriched with isopimaric and sandaracopimaric acids (Fig 3B). In plants subject to weevil feeding, *P*. *pinaster* strongly increased PC1 in the stem, in contrast with a weak induction in *P*. *radiata* (Species × treatment interaction, Table 5A, Fig 3B). No significant changes in the qualitative profile of resin acids (PC2) were observed in response to weevil feeding (Table 5A, Fig 3B). Induced responses in the needles due to weevil damage were negligible in both species (Table 5A, Fig 3B). Pine caterpillar feeding significantly increased PC1 values in the stems of *P*. *pinaster*, but not of *P*. *radiata* (specific contrast tests of control vs induced plants: F_1,18_ = 5.68, *P* = 0.028 and F_1,18_ = 0.01, *P* = 0.911 for each pine species, respectively), with no significant changes in the chemical profile (PC2) in the stems (Table 5B, Fig 3B). In needles, no significant responses to pine caterpillar feeding were detected in *P*. *pinaster*, whereas a marginally non-significant reduction in PC1 (concentration of all resin acids) and a significant decrease for PC2 values (mainly isopimaric and sandaracopimaric concentrations) were found in *P*. *radiata* (Species × treatment interactions, Table 5B, Fig 3B).

**Table 5:** Effects of the pine weevil (a) and pine caterpillar (b), as induction treatments, on the multivariate concentration (PC1) and profile (PC2) of the main resin acids (principal components PC1 and PC2) in the stem and in the needle tissues of two pine species.

	Pine species			Induction treatment			Species × Treatment		
	*Df*	*F*	*P*	*Df*	*F*	*P*	*Df*	*F*	*P*
*a) Pine weevil*									
*Stem*									
PC1	1, 17	26.58	**<0.001**	1, 9	24.9	**<0.001**	1, 17	5.39	**0.033**
PC2	1, 17	17.11	**<0.001**	1, 9	0.04	0.849	1, 17	1.1	0.310
*Needles*									
PC1	1, 18	9.9	**0.006**	1, 9	0.31	0.593	1, 18	0.01	0.939
PC2	1, 18	105.02	**<0.001**	1, 9	1.13	0.315	1, 18	2.54	0.129
*b) Pine caterpillar*									
*Stem*									
PC1	1, 18	24.94	**<0.001**	1, 9	2.39	0.157	1, 18	3.71	0.070
PC2	1, 18	56.74	**<0.001**	1, 9	0.69	0.427	1, 18	0.04	0.839
*Needles*									
PC1	1, 17	10.44	**0.005**	1, 9	1.04	0.335	1, 17	3.7	0.071
PC2	1, 17	50.72	**<0.001**	1, 9	1.86	0.206	1, 17	8.99	**0.008**

Significant p-values (*P* < 0.05) are typed in bold.

## Discussion

Our results showed that inducibility of resin acids in response to the two herbivores was markedly different between the two pine species. Despite those differences, direction of the induction was similar among individual resin acids. In general, *P*. *pinaster* showed high inducibility of resin acids in the stem in response to both insects, whereas *P*. *radiata* lacked evident resin acid-based responses to herbivory, especially against the pine caterpillar, which even caused a significant reduction of the concentration of resin acids in the needles.

Local induced responses in the stem after weevil damage were stronger in *P*. *pinaster* than *P*. *radiata*, supporting prior work performed in the field with these two pine species [56]. Reduced local defensive responses in *P*. *radiata* might subsequently entail greater herbivory damage over time compared to well defended *P*. *pinaster* plants [8, 57], which resulted in pronounced differences in mortality between the two pine species under pine weevil damage [56]. Given that the pine weevil feeds on the cambial tissue, crucial for plant growth and also involved in *de novo* formation of resin ducts that ultimately increase terpene biosynthesis [58, 59], plant survival is expected to be highly contingent on how fast and efficient those induced responses reduce the impact of insect damage in such vital tissues. Nevertheless, recent studies found that gut microbiota of stem chewers can help to overcome host defences by degrading diterpene resin acids if they are at low concentrations [60, 61]. Thus, lower inducibility of resin acid in the stem in response to weevil feeding in *P*. *radiata* compared to *P*. *pinaster* might increase susceptibility to pine weevil damage, as its microbial symbionts can easily degrade resin acids at those levels and increase insect fitness [61].

Systemic induced responses to weevil feeding in the concentration of resin acids in the needles were not found in either of the pine species. Absence of systemic induced responses after stem herbivory for most resin acids was also reported in other pine species [34, 62]. Local induced responses to the pine weevil implied not only *de novo* biosynthesis, but also translocation of defences from distal parts of the plant to the site of injury [63]. It could be possible that systemic induction of defences in the needles might have occurred in response to weevil feeding, but the increase in chemical defences have been compensated by further mobilization of those defences to the site of damage in the stem in both pine species.

Pine caterpillar feeding did not exert significant local induced responses in the needles of *P*. *pinaster*, but substantially reduced resin acid concentration in *P*. *radiata*. The pattern found for total resin acids in the needles was also observed when exploring individual compounds, where no major changes in their concentrations were observed in *P*. *pinaster*, but many resin acids were reduced in *P*. *radiata*. This generalized reduction in the concentration of resin acids of *P*. *radiata* in response to herbivory could be related to what was previously found in other studies, where salivary secretions of caterpillars might compromise induced responses in different host plant species [64–66]. Assuming that this ability also exists for pine caterpillar, herbivore activity may reduce resin acid concentration in the needles at the same time induced responses take place. This might explain why induced responses in the needles were negligible in *P*. *pinaster* after herbivory. In *P*. *radiata*, we can speculate that its limited ability to induce defences plus the putative detoxifying capacity of the pine caterpillar led to the general reduction of resin acid concentration–even below constitutive levels. This strategy probably helps pine caterpillar to reduce the direct and toxic effects of high concentrations of induced resin acids on its fitness.

Systemic induced responses in the stem after pine caterpillar feeding in the needles were only present in *P*. *pinaster*. This pattern was also observed for total polyphenolics in both pine species using the same plant material [9]. This suggests that effectors in the saliva of pine caterpillar may interfere with jasmonate-signalling pathway, favouring systemic rather than local induced responses [67]. Using this strategy, pine caterpillar would force the plant to invest resources to synthesize the costly defensive diterpenes [68] far from the site of attack, and would benefit from avoiding accumulation of defences in the feeding site.

The multivariate characterization of the resin acids shows marked differences in the chemical profiles among tissues and among pine species. These differences in the chemical profile were mainly explained by the relative concentrations of pimaric, sandaracopimaric and isopimaric acids (PC2). *P*. *pinaster* tissues showed higher concentration of pimaric acid, and lower of isopimaric and sandaracopimaric acids, in comparison with *P*. *radiata* tissues that showed the opposite pattern. The multivariate induced responses, however, were similar to univariate responses, mostly relying on quantitative rather than qualitative changes, except for the particular case of *P*. *radiata* against pine caterpillar. Local induced responses in the needles of *P*. *radiata* included both specific changes in the chemical profile, and a general reduction of most resin acids, particularly affecting isopimaric and sandaracopimaric acids. This contrasts with the single reduction in pimaric acid concentration in the *P*. *pinaster*. Pimaric, isopimaric and sandaracopimaric acids were previously observed to strongly and negatively affect herbivory performance, and increase plant resistance [15, 16, 21, 69]. We speculate that pimaric acid might be one of the main target of the herbivore’s molecular patterns, and likely interfered with the suppression and sequestration of other defences by the pine caterpillar. The lack of pimaric acid in the exotic species might have facilitated pine caterpillar to overcome other resin acid-based defences.

Noteworthy, the higher constitutive concentration of resin acids in the needles of *P*. *radiata*, in comparison with *P*. *pinaster*, did not increase resistance to pine caterpillar [47]. Indeed, at field conditions, *P*. *radiata* present higher susceptibility than *P*. *pinaster* to pine caterpillar [70]. This suggests that resistance to herbivory may be also contingent on other traits not measured in this and related previous work [9]. For example, anatomical structures such as stone cells and oxalate crystals depositions are known to be involved in increased resistance to mechanical chewing damage in conifers [58, 71]. Also, intrinsic differences in investment in growth- and defensive-based strategies between both pine species could influence the observed differences in resin acid concentrations and induced responses to herbivory. The exotic *P*. *radiata* exhibits greater growth rates than *P*. *pinaster* [see for example 72, 73], but compensates for lower average terpene levels than the native species [73].

Different stages of plant ontogeny (e.g. seedlings, saplings, and adult trees; current-year vs older leaves) are known to strongly affect variation in constitutive investment and induced responses of terpenes to herbivory. For example, constitutive investment in phloem terpenes was often found to be higher in seedlings and juvenile stages than in adult trees, but the magnitude of induction (i.e. inducibility) was greater in adult trees [74, 75]. In addition, investment in resin acids and phenolic compounds show a strong partitioning between current-year, first-year and older needles, which strongly influences herbivore preference [19, 76], but also tri-trophic interactions [18, 20]. Given that our findings were obtained from one-year-old pines, patterns of induction and timing of defence deployment in response to herbivory in both tissues could differ from those in mature trees and older tissues. In this sense, pines should optimize their investment in defences to deal with the different herbivores with which they often interact throughout the ontogeny. Hence, it is possible that our results reflect ontogenetic matches and mismatches between the pine species and the feeding insects compared to the plant-insect interactions observed in nature, often taking place in very different stages of pine development. In other words, given that the pine weevil mostly feeds on the stem of young conifer seedlings, a strong defensive induction in the stem of young pine seedlings in response to the insect should be expected, which was the case in our study and in previous work with young plants of the same and other conifer species [8, 21, 26, 77]. Conversely, since the pine caterpillar feeds on hosts previously located by the adult moth females using chemical but also visual cues [e.g. apparency, 78] [40, 79, 80], smaller pines, like seedlings, would go unnoticed more often for this insect in natural conditions and, thus, interactions will be unlikely [81]. The lack of plant-pine caterpillar interactions at early stages of development in pines might probably explain why both pine species showed negligible local inducibility in response to this particular pest [see also 9].

## Conclusions

Our study reveals that inducibility of resin acids against insect herbivores is highly localized to the damaged tissue, and stronger in *P*. *pinaster* than in *P*. *radiata*. Also, inducibility was particularly higher in the stem than in the needles. The strong localized induction in response to the pine weevil in the stem contrasts with the very low inducibility of resin acids in the needles in response to the pine caterpillar in both species. Similar patterns were observed when exploring the multivariate profile of resin acids. Given that local inducibility of resin acids in the needles was negligible in both species, and only *P*. *pinaster* showed systemic induction in the stem in response to the pine caterpillar feeding, plant defence suppression mechanisms by the insect, and an ontogenetic mismatch in the seedling-caterpillar interaction for both species might be potentially affecting induced defence deployment against this particular pest. Our findings contribute to the better understanding of the induced responses of diterpene resin acids among pine species against herbivory but future work aimed to assess the potential conflicts of ontogenetic mismatches in plant-insect interactions are needed to comprehend the patterns of inducibility and defensive role of resin acids in both young and mature trees.

## Supporting information

S1 Fig Example of Total Ion Current chromatogram (TIC) of resin acid in elution order (as their methyl esters) (A), and comparison of TIC between undamaged (control) and induced plants by the pine weevil and by the pine caterpillar of two pine species (B) in the stem phloem extracts obtained by gas chromatography and mass spectrometry.
(DOCX)

S2 Fig Chemical structures of the nine resin acids identified by GC-MS (see S1A Fig) and quantified by GC-FID used in this study. (DOCX)
