# Supplementary material for: Resin acids as inducible chemical defences of pine seedlings against chewing insects
Source: PLoS One. 2020 May 1;15(5):e0232692. doi: 10.1371/journal.pone.0232692 (PMC7194405; doi:10.1371/journal.pone.0232692)
Supplement: S1 Fig — Example of Total Ion Current chromatogram (TIC) of resin acid in elution order (as their methyl esters) (A), and comparison of TIC between undamaged (control) and induced plants by the pine weevil and by the pine caterpillar of two pine species (B) in the stem phloem extracts obtained by gas chromatography and mass spectrometry. (DOCX) [file pone.0232692.s001.docx]

**S1 Fig.** Example of Total Ion Current chromatogram (TIC) of resin acid in elution order (as their methyl esters) (A), and comparison of TIC between undamaged (control) and induced plants by the pine weevil and by the pine caterpillar of two pine species (B) in the stem phloem extracts obtained by gas chromatography and mass spectrometry.
