# Supplementary material for: Resin acids as inducible chemical defences of pine seedlings against chewing insects
Source: PLoS One. 2020 May 1;15(5):e0232692. doi: 10.1371/journal.pone.0232692 (PMC7194405; doi:10.1371/journal.pone.0232692)
Supplement: S2 Fig — (DOCX) [file pone.0232692.s002.docx]

**S2 Fig.** Chemical structures of the nine resin acids identified by GC-MS (see Figure A.1) and quantified by GC-FID used in this study. Resin acids are presented in retention order from the upper-left to down-right corners. Levopimaric and palustric acids coeluted during the analytical runs and were treated as a single compound (see Table A.1).
